# Maternal Outcomes of Segmental Resection Versus Cesarean Hysterectomy in Placenta Accreta Spectrum: A Retrospective Comparative Study

**DOI:** 10.7759/cureus.109093

**Published:** 2026-05-18

**Authors:** Engin Korkmazer

**Affiliations:** 1 Obstetrics and Gynecology, University of Health Sciences, Bursa Yüksek İhtisas Training and Research Hospital, Bursa, TUR

**Keywords:** cesarean hysterectomy, gynecology and obstetrics, maternal morbidity, placenta accreta spectrum (pas), segmental uterine resection

## Abstract

Background

Placenta accreta spectrum (PAS) is a life-threatening obstetric condition associated with significant maternal morbidity and mortality. This study aimed to compare maternal and neonatal outcomes between segmental resection and cesarean hysterectomy in patients with PAS.

Methodology

This retrospective case-control study included 130 patients diagnosed with PAS between June 2016 and June 2019. Patients were divided into the following two groups: segmental resection (n = 30) and cesarean hysterectomy (n = 100). The choice of surgical technique was determined based on preoperative imaging findings, intraoperative assessment, and the extent of placental invasion. Demographic characteristics, perioperative findings, and maternal and neonatal outcomes were analyzed.

Results

Patients in the hysterectomy group had significantly higher intraoperative blood loss, longer hospital stay, increased need for blood transfusion, higher rates of bladder injury, longer catheterization duration, and more frequent intensive care unit admission (p < 0.001 for all). No significant differences were observed between groups regarding neonatal outcomes, including gestational age, birth weight, APGAR scores, and neonatal intensive care unit admission.

Conclusions

Segmental resection appears to be a safe and effective alternative to cesarean hysterectomy in selected PAS cases. However, due to potential selection bias and lack of standardized classification, individualized management based on disease severity and intraoperative findings remains essential. Further prospective studies are needed.

## Introduction

Placenta accreta spectrum (PAS) is a life-threatening condition and is associated with maternal mortality and morbidity [1]. PAS is defined as the abnormal invasion of placental tissues into the myometrium [2,3]. With the increased rates of cesarean delivery worldwide, the diagnosis of PAS has increased, and its incidence is known to be 1/500 [4]. PAS risk factors include previous cesarean delivery, placenta previa, previous uterine surgery, and advanced maternal age at delivery [2,5]. Postpartum hemorrhage is the most common cause of maternal death worldwide and a major complication of delivery [6]. The incidence of maternal mortality because of PAS varies between 3% and 10% [7]. Severe postpartum hemorrhage (PPH), need for blood transfusion, disseminated intravascular coagulation (DIC), organ injury, ileus, infection, thromboembolic complications, need for intensive care, renal failure, and increased mortality and morbidity are higher than in uncomplicated pregnancies in PAS cases [8-10]. Cesarean hysterectomy is the most frequently preferred method in the treatment of these cases because of the increased risk of PPH [11]. In PAS cases, the traditional approach is to make an intraoperative diagnosis and then perform a cesarean hysterectomy [2,3,6]. After the recent developments in ultrasonography and magnetic resonance imaging, PAS can be suspected before surgery, and elective surgery can be preferred, leading to favorable maternal outcomes [11,12]. The American College of Obstetricians and Gynecologists (ACOG) recommends cesarean hysterectomy to prevent morbidity and mortality because of severe bleeding because of PAS [1,7]. In PAS, the general approach is delivery by cesarean section, which involves the delivery of the placenta from the uterine cavity after the baby is removed by making a Kerr incision in the uterus [13]. Cesarean hysterectomy is both life-saving and the most effective method for patients with PAS. However, it is technically difficult to perform, especially in patients with bladder invasion, and it causes serious bleeding and loss of fertility [2,5,9]. Cesarean hysterectomy must not be considered a first-line treatment for women who want to be fertile in the future [7]. Recently, fertility-preserving approaches have been tried for patients with PAS, which include leaving the placenta in place, cervical inversion technique, triple P-procedure, and staged cesarean methods [11,13]. Some conservative approaches, such as the Bacri balloon uterine artery, internal iliac artery ligation, and selective arterial embolization, have been described to reduce bleeding mortality and morbidity [2,3,7]. In the present study, local resection of the area with myometrial Invasion was performed with the aim of protecting the uterus [13].

Although the association between PAS and hemorrhage is well established, the novelty of this study lies in the direct comparison of segmental resection and cesarean hysterectomy in a relatively large cohort. The present study aimed to compare obstetric and neonatal outcomes in PAS cases undergoing segmental resection and cesarean hysterectomy.

## Materials and methods

The present study had a retrospective case-control design and included patient data between June 2016 and June 2019. A total of 130 women diagnosed with PAS who gave birth in our clinic were included in the study. First, the patients were divided into the following two groups: segmental resection and cesarean hysterectomy. Segmental resection was performed in 30 patients, and cesarean hysterectomy was performed in 100 patients. PAS was diagnosed by experienced gynecologists and obstetricians based on transvaginal and transabdominal ultrasonography findings, and the diagnosis was confirmed with pathology evaluation. The diagnosis of PAS was established based on preoperative ultrasonographic findings and intraoperative assessment. Histopathological confirmation was available in patients undergoing cesarean hysterectomy and in cases where tissue specimens could be obtained. Diagnostic criteria for PAS on ultrasonography were decreased hypoechogenicity in the retroplacental area, irregular vascular areas in the placenta, and increased vascularity in the myometrium layer between the uterus and bladder [5]. Intraoperative bleeding was defined as bleeding from the beginning of the skin incision to the end of the labor process. Postoperative bleeding was defined as bleeding occurring within the first 24 hours of the postpartum process. The amount of intraoperative bleeding was calculated by taking the amount of blood absorbed by the gauze and the total amount of blood in the aspirator chamber. The decision for transfusion was made by the anesthesiologist, gynecologist, and obstetrician. Factors affecting blood transfusion were preoperative anemia, amount of bleeding during surgery, and hemogram values during the surgery. In the present study, demographic, obstetric, and laboratory data of the patients were scanned from the archive and recorded. Parameters such as surgery duration, amount of transfusion (units), need for cesarean hysterectomy, birth weight, week of birth, 1-5-minute APGAR values, need for neonatal intensive care, and wound infection were also examined.

Surgical modality

Patients with PAS were scheduled for elective cesarean section between 34 and 37 weeks based on the degree of invasion. We preferred to administer general anesthesia to all our patients. All patients’ ureters and bladders were evaluated by postoperative cystoscopy. A midline incision was preferred. After entering the peritoneal cavity, exploration was performed to determine our borders. The baby was removed with a fundal incision, and then the umbilical cord was ligated, and the placenta was left in place. After the fundal incision was sutured, the bilateral hypogastric artery was ligated. After a transverse incision was applied to the uterus, the placenta was separated manually. Resection was performed with scissors and cautery, and bleeding areas were checked. A single layer of continuous suture was employed to close the transverse incision on the anterior uterine wall. Despite this, patients who did not meet the study criteria underwent cesarean hysterectomy.

Following the removal of the fetus and placenta, 20 IU intravenous oxytocin infusion was administered, followed by 15 IU intravenous oxytocin infusion for the first 24 hours. All patients received 2 g of intravenous cefazolin prophylactically, half an hour before the surgery. These patients were mobilized at the sixth hour, and thromboprophylactic therapy was performed.

In the present study, the amount of intraoperative and postoperative bleeding, the amount of transfusion, and the duration of the surgery were evaluated as primary outcomes. Secondary outcomes were evaluated as intraoperative and postoperative complications, length of hospital stay, organ injury, and wound infection.

Patients were included in the study if they had a diagnosis of PAS, established based on prenatal ultrasonographic findings and/or confirmed intraoperatively or by histopathological examination. Eligible patients were those who underwent surgical management in the form of either segmental uterine resection or cesarean hysterectomy. Additionally, only patients who delivered in our tertiary referral center between June 2016 and June 2019 and had complete medical records, including demographic, clinical, and perioperative data, were included. Patients with incomplete or missing medical records, those managed conservatively without surgical intervention, or those subjected to surgical techniques outside the defined study protocol were excluded. Furthermore, patients with insufficient intraoperative or postoperative data and those referred to another center during their management were excluded from the analysis.

Patients were retrospectively identified from the institutional database and divided into the following two groups according to the surgical approach performed: segmental resection and cesarean hysterectomy. Surgical management decisions were not made randomly but were determined based on a combination of preoperative imaging findings, intraoperative assessment, and the extent of placental invasion.

Preoperative evaluation included ultrasonographic examination with Doppler assessment to identify findings suggestive of PAS, including placental lacunae, loss of the retroplacental clear zone, thinning of the myometrium, and abnormal uterovesical interface findings. Ultrasonography with Doppler evaluation was used as the primary diagnostic modality for all patients in accordance with institutional practice.

During surgery, the extent and localization of placental invasion, the degree of myometrial involvement, the presence of suspected surrounding tissue involvement, and the feasibility of preserving uterine integrity were evaluated. Segmental resection was considered in selected patients with localized invasion where fertility preservation was considered feasible and surgically safe. Cesarean hysterectomy was preferred in cases with extensive placental invasion, diffuse uterine involvement, uncontrolled hemorrhage, or when fertility-preserving surgery was considered unsuitable.

Demographic characteristics, obstetric history, perioperative findings, maternal outcomes, and neonatal outcomes were extracted from medical records and comparatively analyzed between the study groups.

Statistical analysis

SPSS version 21.0 (IBM Corp., Armonk, NY, USA) was employed for all statistical analyses. Descriptive variables were expressed as mean ± standard deviation or median (minimum: maximum) values. The Student’s t-test and Mann-Whitney U test were employed to define variables between groups, and p-values ≤0.05 were considered statistically significant.

Ethical considerations

The study was approved by the Ethics Committee of Bursa Health Sciences University High Specialization Training and Research Hospital, a tertiary hospital, with the ethics committee number 2011-KAEK-25 2019/08-14. This study was conducted following the Declaration of Helsinki principles.

## Results

The clinical and demographic characteristics of the study population are presented in Table 1. Patients in the local resection group were younger than those in the hysterectomy group (31.0 ± 5.3 vs. 33.6 ± 4.8; p = 0.001). Gravida and parity were more frequent in patients who underwent hysterectomy (p < 0.001, p < 0.001, p < 0.001). The history of delivery by cesarean section was similar in both groups (p = 0.229), whereas there was less history of myomectomy in the local resection group (p < 0.001).

**Table 1:** Demographic and clinical characteristics of patients.

Variable	Segmental resection (n = 30)	Cesarean hysterectomy (n = 100)	P-value
Age (years)	31.0 ± 5.3	33.6 ± 4.8	0.001
Gravida	2.9 ± 1.7	4.3 ± 1.9	<0.001
Parity	1.7 ± 0.2	3.0 ± 0.1	<0.001
Body mass index (kg/m²)	24.5 ± 2.5	25.0 ± 2.0	0.115
≥1 Abortion, n (%)	4 (13.3)	22 (21.5)	0.226
≥3 Cesarean, n (%)	4 (13.3)	23 (23.0)	0.229
History of myomectomy, n (%)	19 (63.3)	86 (85.9)	<0.001

Perioperative data and maternal outcomes are presented in Table 2. The amount of bleeding during surgery, hospital stay, use of intra-abdominal drain, bladder injury, postoperative catheterization time, intensive care stay, and intraoperative and postoperative transfusion amounts were higher in the hysterectomy group (p < 0.001 for all).

**Table 2:** Perioperative data and maternal outcomes.

Variable	Segmental resection (n = 30)	Cesarean hysterectomy (n = 100)	P-value
Preoperative hemoglobin (g/dL)	9.8 ± 1.7	9.6 ± 1.3	0.567
Postoperative hemoglobin (g/dL)	8.9 ± 1.4	9.0 ± 1.3	0.894
Blood loss (mL)	877 ± 62	1,157 ± 133	<0.001
Hospital stay (days)	5.2 ± 0.3	7.7 ± 0.2	<0.001
Operation time (minutes)	100.5 ± 13.1	97.1 ± 15.6	0.118
Bladder injury, n (%)	3 (10.0)	35 (35.0)	<0.001
Intestinal injury, n (%)	3 (10.0)	8 (8.0)	0.684
Catheter duration (days)	2.0 ± 0.3	4.0 ± 0.4	<0.001
Intraoperative transfusion, n (%)	15 (50.0)	98 (98.0)	<0.001
Postoperative transfusion, n (%)	13 (43.3)	77 (77.0)	<0.001
Intensive care unit admission, n (%)	2 (6.7)	27 (27.0)	<0.001
Reoperation, n (%)	1 (3.3)	6 (6.0)	0.420
Wound infection, n (%)	4 (13.3)	14 (14.0)	0.685

Neonatal results are presented in Table 3. No significant differences were detected between the two groups in terms of gestational age at birth, birth weight, one-minute APGAR score, five-minute APGAR score, and neonatal intensive care need (p = 0.058, p = 0.034, p = 0.076, p = 0.0062, p = 0.131).

**Table 3:** Neonatal outcomes.

Variable	Segmental resection (n = 30)	Cesarean hysterectomy (n = 100)	P-value
Gestational age (weeks)	35.2 ± 3.9	32.1 ± 1.3	0.058
Birth weight (g)	2582 ± 87	2555 ± 242	0.034
1-minute APGAR score	8.9 ± 0.2	8.8 ± 0.4	0.076
5-minute APGAR score	9.2 ± 0.2	9.7 ± 0.5	0.062
Neonatal intensive care unit admission, n (%)	5 (16.7)	9 (9.0)	0.131

## Discussion

PAS disorder is a difficult condition that can have serious obstetric consequences for both surgeons and patients. Its frequency is rapidly increasing today because of the increase in previous cesarean sections. ACOG recommends early cesarean hysterectomy with the placenta left in situ to reduce complications that may occur because of massive hemorrhage in PAS cases. However, the committee also states that the management plan for each case should be individualized.

When the literature was reviewed, there were many conservative methods. The aim was not only to protect fertility but also to prevent women from being stigmatized as having lost their femininity in society. It was aimed to ensure that women are better both psychologically and in terms of their quality of life. When the segmental resection method we employed in the present study was compared with hysterectomy, complications were fewer in segmental resection. When the studies in the literature were examined, morbidity could be reduced in suitable cases, and the patient’s uterus could be protected [14,15]. When previous studies were examined, the need for transfusion in PAS cases could be up to 46.9%, and the total amount of bleeding could be up to 5,000 mL. In PAS cases, where the risk of bleeding and transfusion is already very high, it was shown both in the present study and other studies in the literature that the amount of bleeding could be less with segmental resection [16,17]. Another complication in PAS cases is urinary system injuries. When the studies in the literature were examined, although there is no statistical comparison, this rate increases up to 31% in cesarean hysterectomy. However, it was seen that this rate was 14% in conservative methods. In the present study, the statistical analysis showed that urinary system injury was significantly higher in the cesarean hysterectomy group. For this reason, the selection of segmental resection in appropriate PAS cases provides advantages against both bleeding and urinary system injuries [17-20].

When PAS risk factors were examined, the importance of previous uterine surgery was known. When the results of the present study are examined, it is seen that a history of myomectomy among previous uterine surgeries increases the risk of hysterectomy. For this reason, we think that the patient’s myomectomy history can be a guide in choosing segmental resection or hysterectomy in PAS cases.

In PAS cases, the need for intensive care may be inevitable because of massive bleeding and complications. In line with the literature, the need for intensive care is higher in the cesarean hysterectomy group in the present study [20-25]. Another important point was that no difference was detected between the two groups in terms of resurgery.

When the neonatal outcomes were evaluated for both patient groups in the present study, no statistical difference was found between the two groups. For this reason, segmental resection in suitable patients and with surgical experience will not lead to a negative neonatal outcome.

Several limitations of this study should be considered. First, the retrospective design may have introduced selection bias and limited control over confounding factors. Second, this was a single-center study, which may affect the generalizability of the findings to different populations and clinical settings. Although the sample size was acceptable for the study population, larger prospective multicenter studies are needed to validate these findings. In light of the results in the present study, we think that the reason for less morbidity in segmental resection is that surgeons do not prefer to perform segmental resection in PAS cases without gaining sufficient experience. Moreover, lower-grade PAS cases may have been preferred for segmental resection because of the surgeons’ experience and clearer examination of the placenta in the preoperative period, and we think that the fact that cesarean hysterectomy involves a more comprehensive procedure also plays a role.

In the present study, the preferability of segmental resection in PAS cases was examined. When the results were examined, morbidity was less in cases with segmental resection compared to cesarean hysterectomy [25].

## Conclusions

The findings of this study indicate that local resection may represent a safe and feasible surgical option in carefully selected patients with PAS. In particular, favorable outcomes appear to be associated with appropriate patient selection based on the degree of placental invasion and the absence of extensive extrauterine involvement. Furthermore, the implementation of a standardized multidisciplinary management protocol, including preoperative planning, intraoperative hemostatic strategies, and experienced surgical teams, may contribute to a reduction in perioperative complications, blood loss, and the need for hysterectomy. These approaches have the potential to decrease both maternal morbidity and mortality while preserving fertility in selected cases.
